# RAB11FIP1: An Indicator for Tumor Immune Microenvironment and Prognosis of Lung Adenocarcinoma from a Comprehensive Analysis of Bioinformatics

**DOI:** 10.3389/fgene.2021.757169

**Published:** 2021-10-26

**Authors:** Wenyi Zhang, Ting Chen, Jun Liu, Shali Yu, Lei Liu, Miaosen Zheng, Yifei Liu, Hongbing Zhang, Tingting Bian, Xinyuan Zhao

**Affiliations:** ^1^ School of Public Health, Nantong University, Nantong, China; ^2^ Department of Pathology, Affiliated Dongtai Hospital of Nantong University, Dongtai, China; ^3^ The Sixth Affiliated Hospital, Sun Yat-Sen University, Guangzhou, China; ^4^ Department of Pathology, Affiliated Hospital of Nantong University, Nantong, China; ^5^ Jiangsu Provincial Center for Disease Control and Prevention, Nanjing, China

**Keywords:** RAB11FIP1, lung adenocarcinoma, cancer immune infiltrates, prognosis, bioinformatic analysis

## Abstract

Lung adenocarcinoma (LUAD) was the first one all over the world. RAB11FIP1 was found to be expressed differently in a critical way among different cancers. However, the prognostic value and immune infiltration of RAB11FIP1 expression in LUAD are unclear. In this study, the expression of RAB11FIP1 in LUAD was investigated in the Oncomine, TCGA, GEO, and UALCAN databases. Kaplan-Meier analysis was chosen to compare the association between RAB11FIP1 expression and overall survival (OS) in LUAD patients. The dataset of TCGA was used to analyze the pertinence between RAB11FIP1 and clinicpathological factors. GO, KEGG, and network analysis of protein-protein interactions (PPI) were conducted to investigate the potential mechanism of RAB11FIP1. In the end, the relevance of RAB11FIP1 to cancer-immune infiltrates was investigated. RAB11FIP1 was found to be down-regulated by tumors compared with adjacent normal tissue in multiple LUAD cohorts. RAB11FIP1 is an independent prognostic factor in lung adenocarcinoma. There was a high correlation between low RAB11FIP1 in tumors and worse OS in LUAD. Functional network analysis suggested that RAB11FIP1 was associated with multiple pathways. Besides, the expression of RAB11FIP1 was closely related to the infiltration levels of B cell, CD8^+^ T cells, CD4^+^ T cells, macrophages, neutrophils, and dendritic cells. RAB11FIP1 expression in LUAD occurred with a variety of immune markers. Our findings suggest that RAB11FIP1 is related to prognosis and immune infiltrates in LUAD.

## Introduction

Lung cancer is a major type of cancer, causing death all over the world (Nasim et al., 2019), with over 40% of cases being lung adenocarcinoma (LUAD) (Jiang et al., 2020). At present, the treatment for LUAD mainly includes surgery, radiotherapy, and chemotherapy. For those seriously ill patients, medication is ineffective. To improve patient survival, the identification of prognostic markers and effective drug targets are urgently needed (Zhang et al., 2020).

In recent years, studies on various cancer cell-extrinsic regulators in the tumor microenvironment (TME) have come into researchers’ field of vision (Bianchini et al., 2020). Many studies have suggested that components of cells in the TME play a regulatory role in tumor proliferation, angiogenesis, invasion and metastasis, and chemotherapeutic resistance, as well as other hallmarks of cancers (Lee and Cheah, 2019). In the tumor microenvironment, tumor-infiltrating immune cells (TIICs) regulate cancer progression and exert potential prognostic value (Zhang et al., 2019). Since TIICs are appealing therapeutic targets, the discovery of more immune targets or undiscovered immune mechanisms is essential for TIICs investigation.

RAB11FIP1 is a member of Rab11 family interacting proteins (Rab11-FIPs). RAB11FIP1 binds to Rab11 through a carboxyl-terminal amphipathic alpha-helix (Jin and Goldenring, 2006). Growing evidence suggests that dysregulation of Rab11-FIPs causes various pathophysiological diseases including cancer (Cho and Lee, 2019). Some studies suggested that RAB11FIP1 over-expression leads to breast cancer progression and RAB11FIP1 might be potential therapeutic targets for cervical cancer and its precursors (Zhang et al., 2016; Takao et al., 2017). Nevertheless, the potential effects of RAB11FIP1 in LUAD and the relationship between RAB11FIP1 and TIICs are doubtful.

In this study, we observed that low levels of RAB11FIP1 were associated with unfavorable clinical outcomes in LUAD. RAB11FIP1 was significantly related to overall survival (OS) in LUAD patients. RAB11FIP1 is an independent prognostic indicator. The probable pathways of RAB11FIP1 were ascertained via PPI network analysis. A new lncRNA-miRNA-mRNA ceRNA network that may influence the lung cancer progression was constructed. Furthermore, our research uncovered a potential link between tumor immunity and the expression of RAB11FIP1. In short, our study suggested that the RAB11FIP1 had important prognostic value and immune correlation in LUAD.

## Materials and Methods

### Oncomine and Human Protein Atlas Database Analyses

The mRNA expression levels and DNA copy number of RAB11FIP1 in LUAD were analyzed using Oncomine 4.5 database, a maximal oncogene chip database and an integrated data-mining platform (Liang et al., 2020). Our data came from the Hou Lung (Hou et al., 2010), Selamat Lung (Selamat et al., 2012), Landi Lung (Landi et al., 2008), Su Lung (Su et al., 2007), Garber Lung (Lin et al., 2016), and TCGA Lung datasets. The differences in RAB11FIP1 protein levels between tumor and normal tissues were analyzed using the Human Protein Atlas (HPA) website (Lánczky et al., 2016).

### UALCAN Analysis

UALCAN, an interactive web portal including TCGA level 3 RNA-seq and clinical data from 31 cancer types, analyzes TCGA gene expression data in more depth (Chandrashekar et al., 2017). In this study, UALCAN was applied to compare the transcription level of RAB11FIP1 in lung cancer tissues and normal samples, as well as the transcription level of RAB11FIP1 in disparate sub-types and sub-stages. Our research included all available lung cases on UALCAN.

### TCGA and Gene Expression Omnibus Database Analysis

Gene expression profile and the related clinical data from a total of 508 tumor samples and 44 normal samples of patients with LUAD were downloaded from TCGA. Defective clinical samples were deleted. Then paired sample analysis was conducted and the connection between gene expression and clinicopathological parameters as well as OS was examined. Univariate and multivariate Cox analyses were made according to the data from TCGA and GEO (GSE72094). Gene set enrichment analysis (GSEA) was utilized along with GO and KEGG annotation to assess whether RAB11FIP1 showed statistically significant, concordant differences between tumor samples and normal samples (Subramanian et al., 2007). False discovery rate (FDR) less than 0.05 and nominal *p* value less than 0.05 were deemed as statistical significance. In addition, the dataset was separated into two groups based on the median RAB11FIP1 expression levels. Finally, the immune scores of 28 types of TIICs were measured via the CIBERSORT algorithm and the connection between the expression of RAB11FIP1 and TIICs was assessed (Newman et al., 2015). All of the data were analyzed by R software (version 4.0.4) and Strawberry Perl.

### Survival Analysis by Kaplan-Meier Plotter

Kaplan-Meier (KM) Plotter is a survival analysis tool tailored for medical research. The prognostic values of RAB11FIP1 at mRNA level were analyzed in lung cancer. All the lung cancer samples from KM Plotter were included, and hazard ratio (HR), 95% confidence interval (95% CI), and log-rank *p* value were calculated and analyzed.

### LinkedOmics Analysis

The LinkedOmics database (http://www.linkedomics.org) was selected to analyze multi-omics data and clinical data from 32 cancer types and 11,158 patients (Vasaikar et al., 2018). The LinkFinder module of LinkedOmics showed that differentially expressed genes (DEGs) were in correlation with RAB11FIP1 in the LUAD cohort (n = 515, ID-80884). All results were analyzed by Pearson’s correlation coefficient and were demonstrated in heat maps. The LinkInterpreter module identified pathways and networks composed of DEGs. The data from the LinkFinder were signed and ranked, and GSEA was performed to elucidate GO (CC, BP, and MF), KEGG pathways. The rank criterion was FDR <0.05 and 500 simulations were performed.

### Tumor Immunity Estimation Resource Database Analysis

TIMER (https://cistrome.shinyapps.io/timer/) is a comprehensive resource for analysis of tumor-immune interactions, including 10,897 samples of 32 types of cancer (Li et al., 2017). TIMER uses a deconvolution algorithm to infer the abundance of TIICs from gene expression profiles (Li et al., 2016). The correlation between the copy number variation (CNV) of RAB11FIP1 and the abundance of six types of TIICs (including B cells, CD4^+^ T cells, CD8^+^ T cells, neutrophils, macrophages, and dendritic cells) were explored. Then, a correlation module was used to evaluate the correlation between RAB11FIP1 expression and immune cell marker genes. These genes are provided by the website.

### Gene Expression Profiling Interactive Analysis Database Analysis

Gene Expression Profiling Interactive Analysis (GEPIA) is an online database making gene expression profiling and interactive analyses with cancer and normal samples (Tang et al., 2017). In this study, GEPIA was used to verify the link discovered by TIMER between RAB11FIP1 and immune marker genes. Spearman correlation statistical method was used to calculate the correlation coefficient.

### Protein Interactions Network and Module Analysis

The Search Tool for the Retrieval of Interacting Genes (STRING) database is a database of predicted functional associations between proteins containing 261,033 orthologs in 89 fully sequenced genomes (Mering et al., 2003). The co-expressed genes from the cBioPortal database were achieved and were put into STRING to construct the PPI network (Gao et al., 2013). The results were visualized using Cytoscape software. Whereafter, the two most important modules were selected and subjected the genes to functional enrichment analysis.

### Statistical Analysis

All statistical analyses were performed in the Bioinformatics Online Database and R (version 4.0.4). The differential expression of RAB11FIP1 mRNA in LUAD tissues was analyzed by Student’s t-test. Kaplan-Meier curves were used to compare the OS among different groups. The log-rank test was performed to indicate the significance of survival time differences. The data from TCGA and NCBI were performed by R programming of the package. The Kruskal test was applied to inspect the relationship between RAB11FIP1 expression and sub-types. The correlation between RAB11FIP1 expression and lymph node status and distant metastasis was assessed using the Wilcox test. Furthermore, logistic regression analysis was conducted to assess the relevance of the gene expression to clinicopathological parameters. Univariate and multivariate Cox analyses were adopted to evaluate the value of the RAB11FIP1 gene as a prognostic indicator. Spearman’s correlation coefficient revealed how closely related different genes were. The threshold of *p* < 0.05 indicated the significance of correlation applies to all analyses.

## Results

### RAB11FIP1 Expression Levels in Lung Adenocarcinoma

**FIGURE 1:**
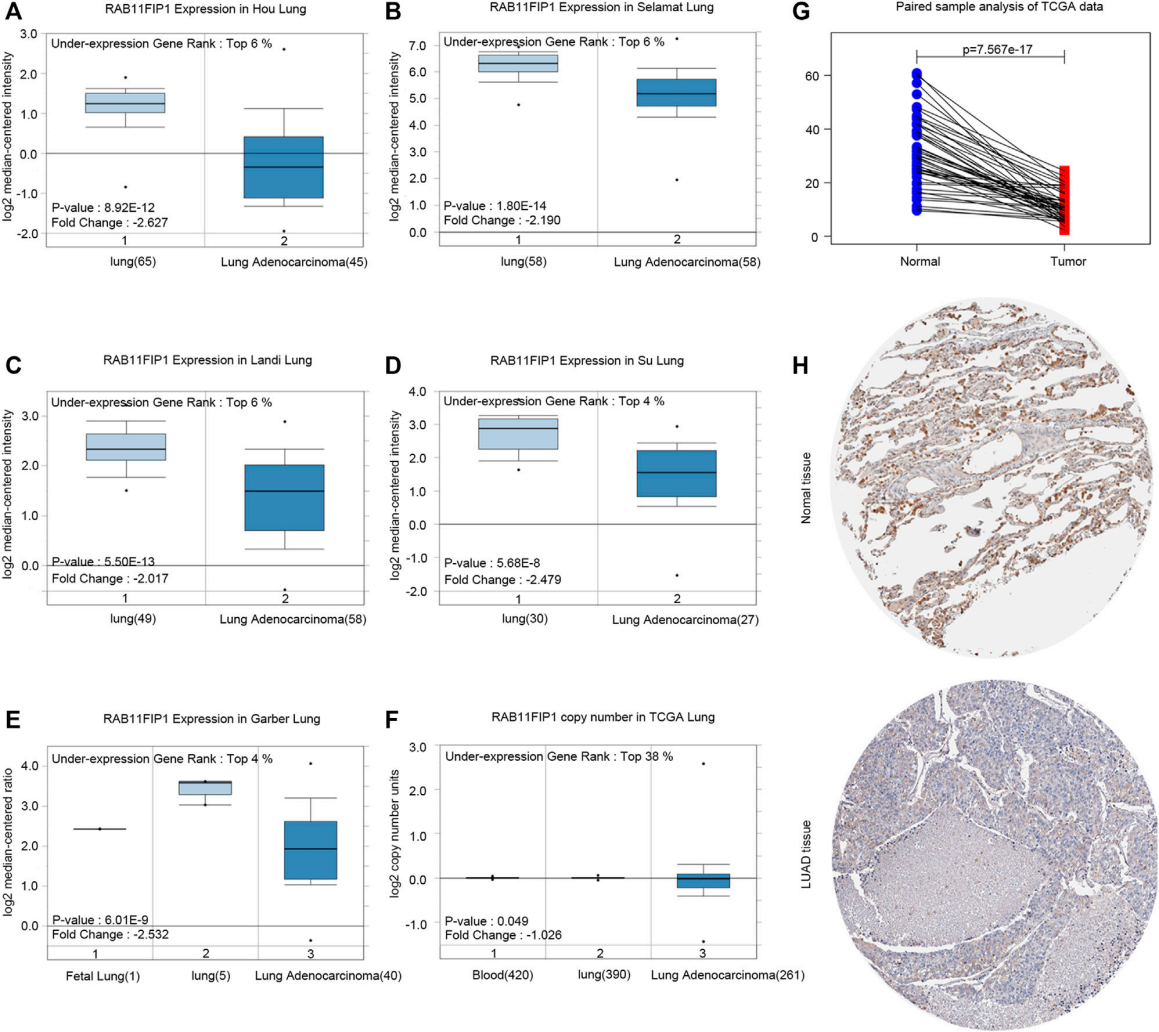
RAB11FIP1 expression levels in LUAD. **(A–E)** Box plot showing RAB11FIP1 mRNA levels in Hou Lung, Selamat Lung, Landi Lung, Su Lung, Garber Lung datasets. **(F)** Box plot showing RAB11FIP1 copy number in TCGA Lung datasets, respectively. **(G)** The paired expression of RAB11FIP1 between normal and tumor tissues. **(H)** RAB11FIP1 protein levels in normal lung and LUAD were visualized by IHC in HPA.

RAB11FIP1 transcription levels in several LUAD studies from TCGA and the Gene Expression Omnibus (GEO) were firstly analyzed and the data of the Oncomine4.5 database showed that mRNA expression levels and DNA CNV of RAB11FIP1 in LUAD were dramatically lower than that of normal tissue (Figures 1A–F). Paired sample analysis of TCGA data was also conducted, and the results confirmed the above conclusions (Figure 1G). Consistently, immunohistochemical data from the HPA database showed the RAB11FIP1 protein level in LUAD tissue was significantly down-regulated as well (Figure 1H).

### Association of RAB11FIP1 Expression Levels With the Clinical Characteristics

**FIGURE 2:**
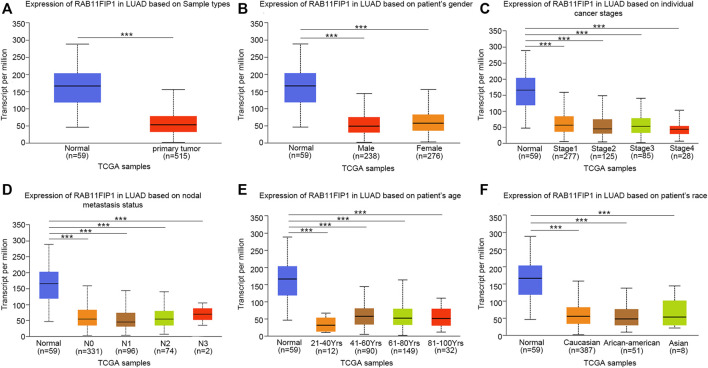
Transcription of RAB11FIP1 stratified by gender, age, and other criteria in a subgroup of patients with lung cancer. **(A)** Box-plot showing the relative expression of RAB11FIP1 in normal and lung cancer samples. **(B)** Box-plot showing the relative expression of RAB11FIP1 in normal individuals and male or female patients with lung cancer. **(C)** Box-plot showing the relative expression of RAB11FIP1 in normal subjects or patients with stage 1, 2, 3, or 4 lung cancers. **(D)** Box-plot showing the relative expression of RAB11FIP1 in normal individuals or patients with metastatic tumors. **(E)** Box-plot showing the relative expression of RAB11FIP1 in healthy subjects of any age and patients with lung cancer aged 21–40, 41–60, 61–80, and 81–100 years **(F)** Box-plot showing the relative expression of RAB11FIP1 in normal and LUAD samples based on the ethnicity of patients. *, *p* < 0.05, ***, *p* < 0.001.

Furthermore, the sub-group analysis of various several pathologic characteristics of LUAD samples was performed using the UALCAN database, and the RAB11FIP1 transcription decreased consistently. The sub-group analysis on gender, disease stage, tumor metastasis, age, and ethnicity showed that the expression of RAB11FIP1 of LUAD patients was significantly lower than that in the control group (Figure 2). Logistic regression analysis results indicated that RAB11FIP1 expression at stage II was lower than that at stage I (*p* = 0.012), that in T2 was lower than that in T1 (*p* = 0.031), and that in T3 was lower than that in T1 (*p* < 0.001). On top of that, RAB11FIP1 expression had nothing to do with gender, age, positive lymph node metastasis vs the negative, or positive distant metastasis versus negative (Table 1).

**TABLE 1:** Association between RAB11FIP1 expression and clinicopathologic parameters by Logistic regression.

Clinical parameters	Total(N)	Odds ratio in RAB11FIP1 expression	*p*-Value
Gender (male vs female)	477	0.730 (0.508–1.048)	0.089
Stage (II vs I)	366	0.561 (0.355–0.880)	0.012
Tumor size (T2 vs T1)	414	0.644 (0.431–0.960)	0.031
Tumor size (T3 vs T1)	455	0.286 (0.132–0.589)	0.001
Lymph node metastasis (positive vs negative)	474	0.756 (0.467–1.218)	0.254
Distant metastasis (positive vs negative)	348	0.697 (0.293–1.603)	0.400

### The Expression Level of RAB11FIP1 Was Associated With Survival

**FIGURE 3:**
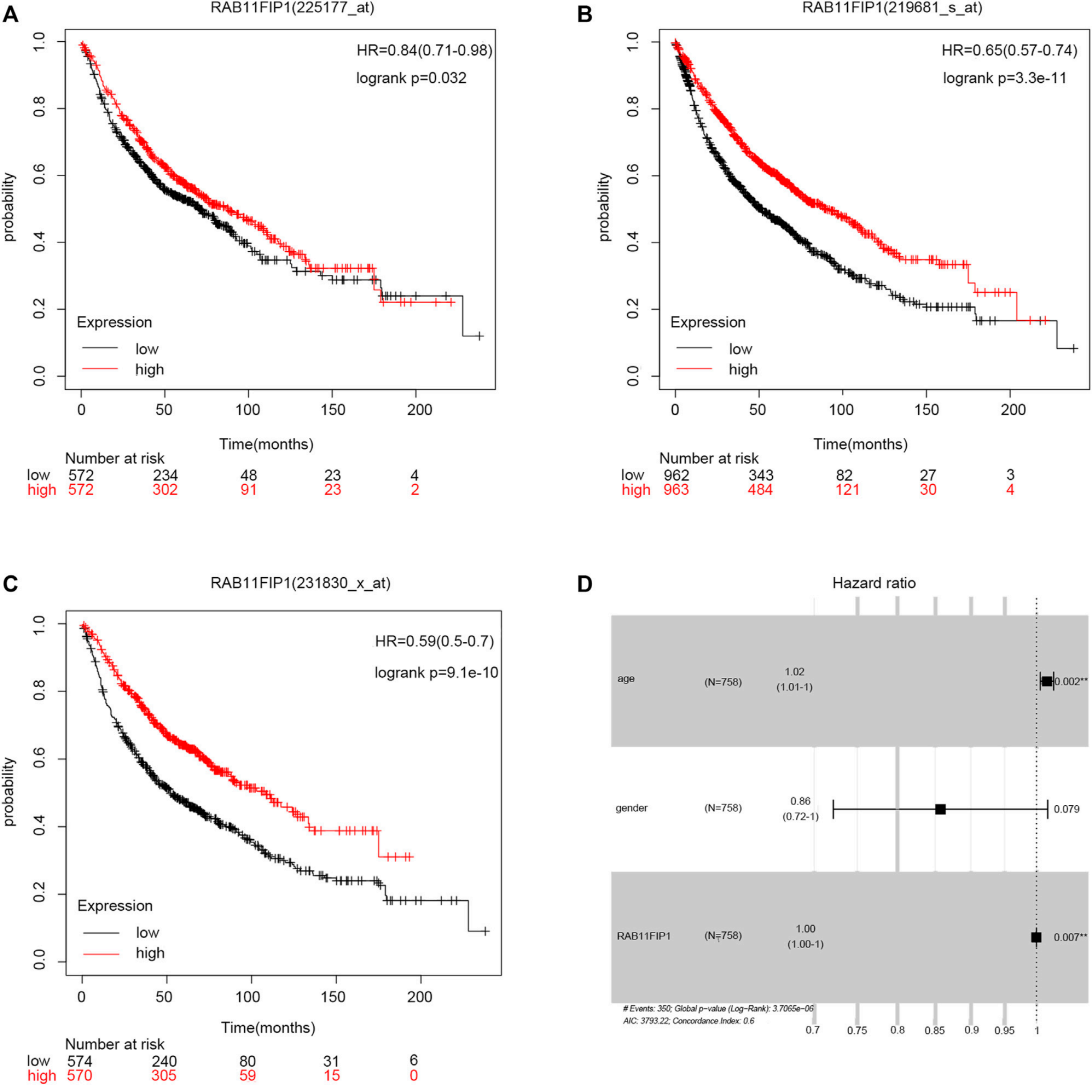
Relationship between RAB11FIP1 and prognosis in LUAD. **(A–C)** Survival curve of differential RAB11FIP1 expression in LUAD. **(D)** Multivariate Cox analysis of RAB11FIP1 expression and other clinicopathological variables.

In this study, Kaplan-Meier survival curves were used for evaluating the association of RAB11FIP1 expression level with survival rates in the three LUAD cohorts (A: 225177_at, B: 219681 s_at, C: 231830 s_at) that were available from the database (Figures 3A–C). Patients were divided into two groups based on the median expression level of RAB11FIP1. The results demonstrated that the overall survival (OS) of patients with lung cancer in the low RAB11FIP1 expression group compared with those with the high RAB11FIP1 expression group was significantly shorter (log-rank test, *p* < 0.05). RAB11FIP1 expression was significantly related to OS in patients with lung cancer and might be considered as a promising biomarker for predicting survival in patients with LUAD. As shown in the forest plot (Figure 3D), the multivariate analysis showed that RAB11FIP1 (HR = 0.9997, *p* = 0.0069) had an independent prognostic value (Table 2).

**TABLE 2:** Univariate and multivariate Cox analysis of clinicopathological parameters and OS in patients with LUAD.

Clinicopathologic parameters	Univariate analysis		Multivariate analysis	
	HR (95%CI)	*p*-value	HR (95%CI)	*p*-value
age	1.0178(1.0070–1.0287)	0.0011	1.0169(1.0061–1.0278)	0.0021
gender	0.7679(0.6535–0.9023)	0.0013	0.8572(0.7218–1.0181)	0.0791
RAB11FIP1	0.9996(0.9995–0.9998)	0.0002	0.9997(0.9995–0.9999)	0.0069

### Gene Set Enrichment Analysis

**FIGURE 4:**
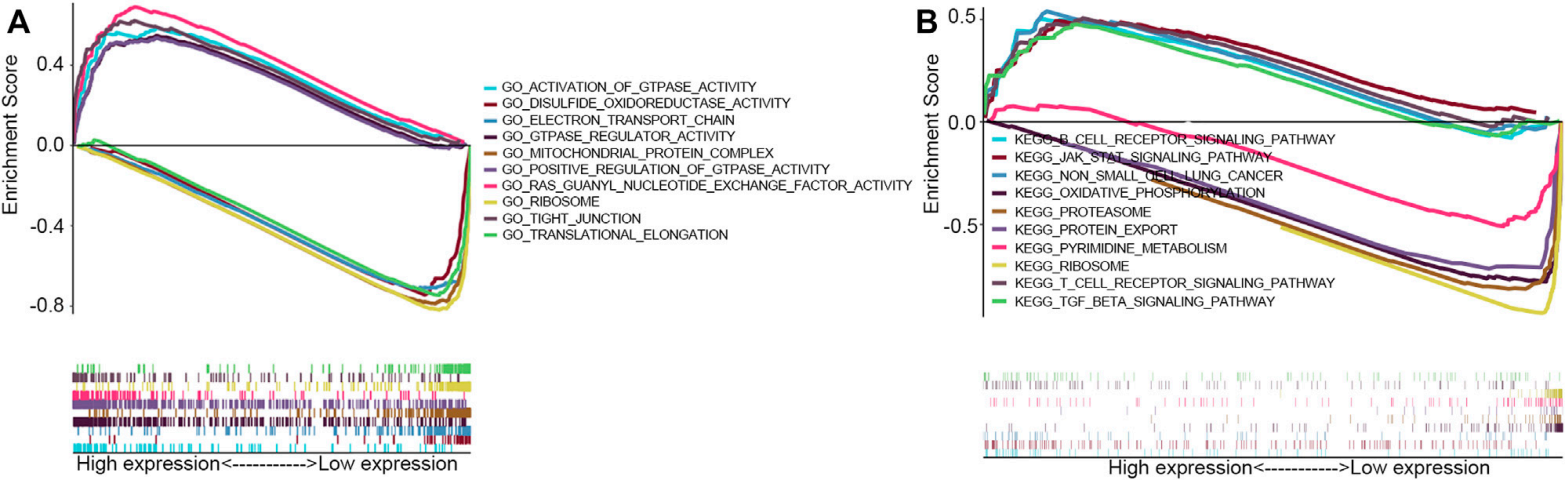
GSEA with GO term and KEGG pathway. **(A)** GO terms analysis revealed the most five positively correlated pathways and five most negatively correlated pathways. **(B)** KEGG pathways analysis showed the most five positively correlated pathways and five most negatively correlated pathways.

GO term and KEGG pathway by gene set enrichment analysis (GSEA) was performed to elucidate the biological function of RAB11FIP1 expression. The filter criteria was that enrichment score | NSE | > 1 (*p* < 0.05), and according to which, the five most relevant signal pathways were selected. GO term analysis indicated that tight junction, Ras guanyl nucleotide exchange factor activity, activation of GTPase activity, GTPase regulator activity, and positive regulation of GTPase activity were most positively related to the expression of RAB11FIP1. The disulfide oxidoreductase activity, electron transport chain, mitochondrial protein complex, ribosome, and translational elongation were most negatively related to the expression of RAB11FIP1 (Figure 4A). KEGG pathway analysis indicated the following five most correlative pathways, namely non-small cell lung cancer, JAK-STAT signaling pathway, T cell receptor signaling pathway, TGF-beta signaling pathway, and B cell receptor signaling pathway. The five most negatively correlative pathways included oxidative phosphorylation, protein export, proteasome, ribosome, and pyrimidine metabolism (Figure 4B).

### Enrichment Analysis of the Co-Expressed Genes

**FIGURE 5:**
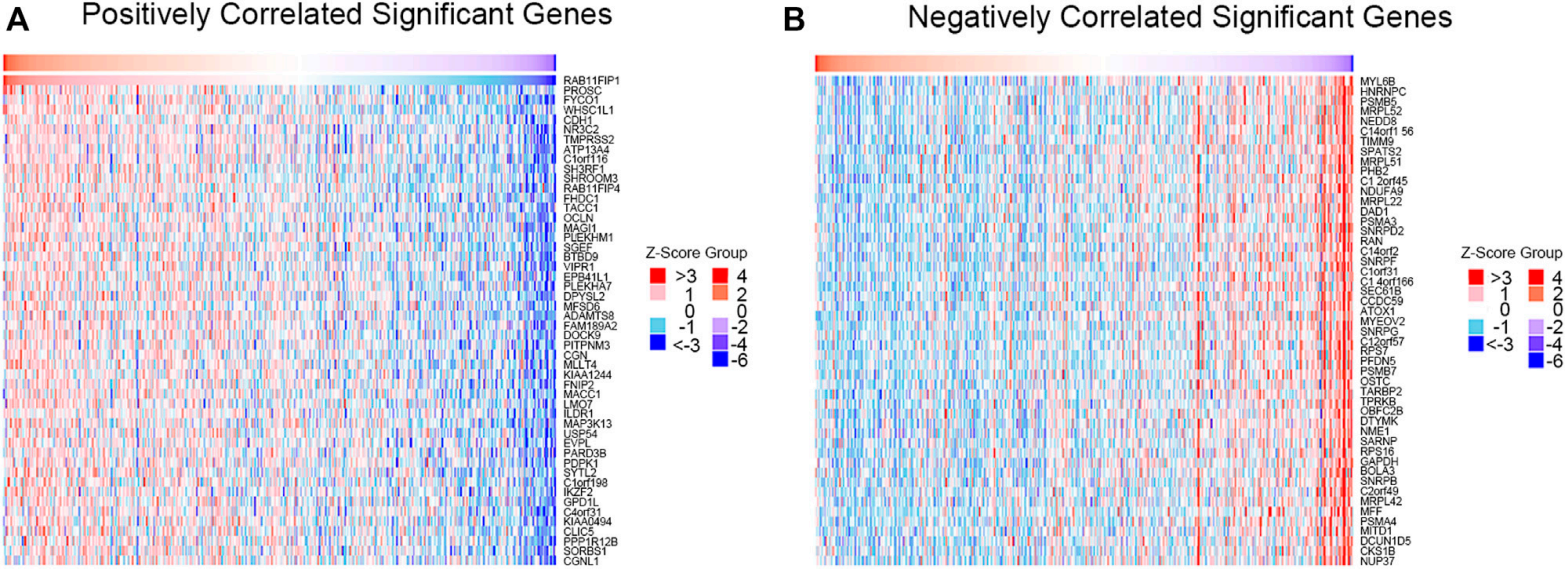
Correlation between gene differential expression and RAB11FIP1 in LUAD. **(A, B)** Heat maps show positive and negative genes associated with RAB11FIP1 in lung cancer (top 50). Red is a positive gene correlation; green is a negative gene correlation.

**FIGURE 6:**
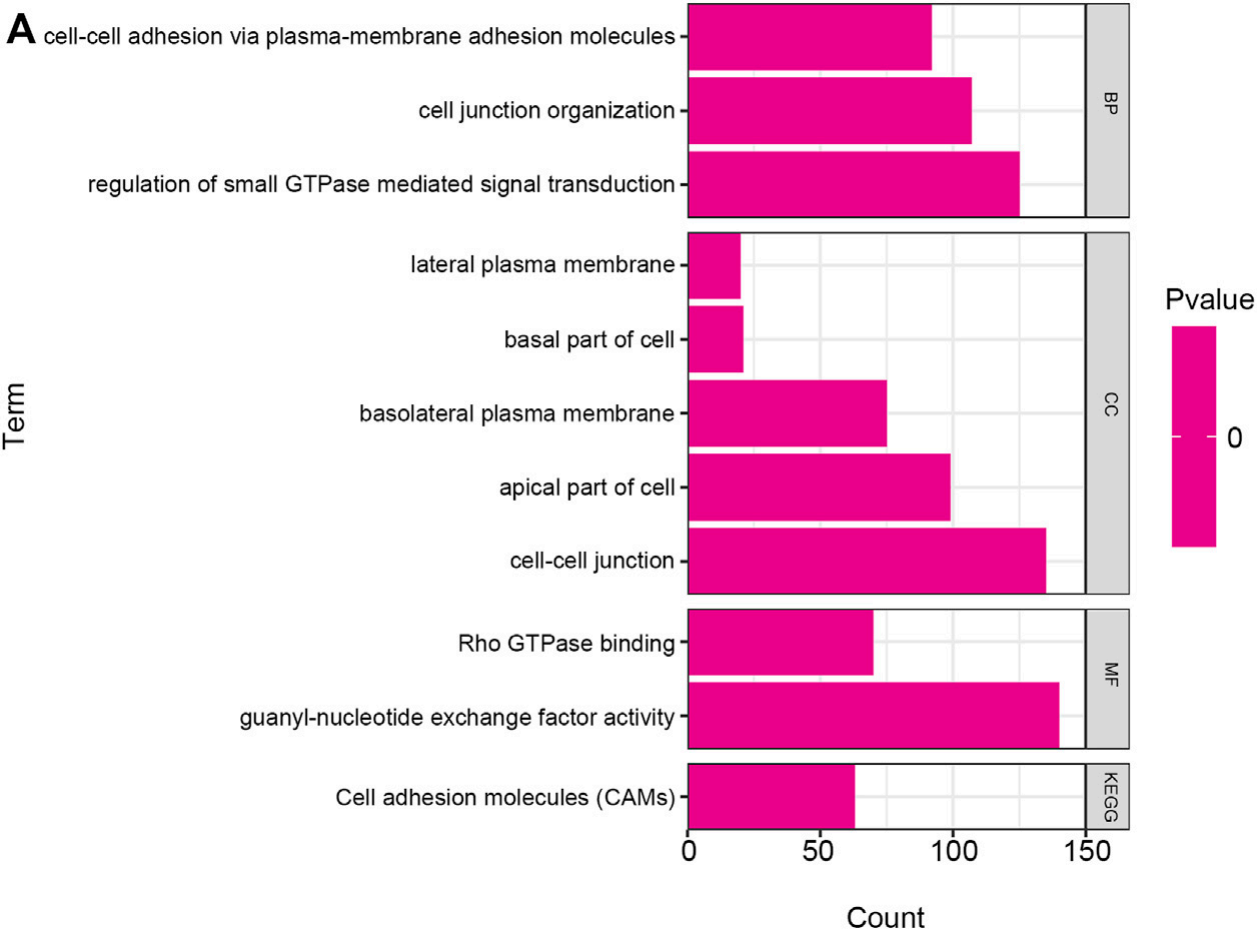
GO annotation and KEGG pathway in lung cancer. The GO annotation and KEGG pathway of RAB11FIP1 co-expressed genes in lung tissue were analyzed by GSEA. (A) Biological processes. Cellular components. Molecular functions. KEGG pathway analysis. FDR from GSEA was 0.

GO and KEGG pathways co-expression analysis of RAB11FIP1 related genes in lung cancer mRNA sequencing data with 515 patients from the TCGA were analyzed using the functional module of Linkedomics. As shown in the heat map, the top 50 marked genes were positively and negatively related to RAB11FIP1 (Figures 5A,B). These results implied that RAB11FIP1 influenced the transcriptome extensively. GO analysis in GSEA showed the correlation between gene differential expression and RAB11FIP1. They were primarily involved in the regulation of small GTPase mediated signal transduction, cell junction organization, and cell-cell adhesion via plasma-membrane adhesion molecules. It was mainly located in the cell-cell junction, basolateral plasma membrane, lateral plasma membrane, apical part of the cell, and basal part of the cell. Their molecular function was related to guanyl-nucleotide exchange factor activity, Rho GTPase binding. KEGG pathway analysis displayed the enrichment pathway of cell adhesion molecules (Figure 6 and Supplementary Table S1–S4).

### Construction of Protein-Protein Interactions Network of Co-Expressed Genes

**FIGURE 7:**
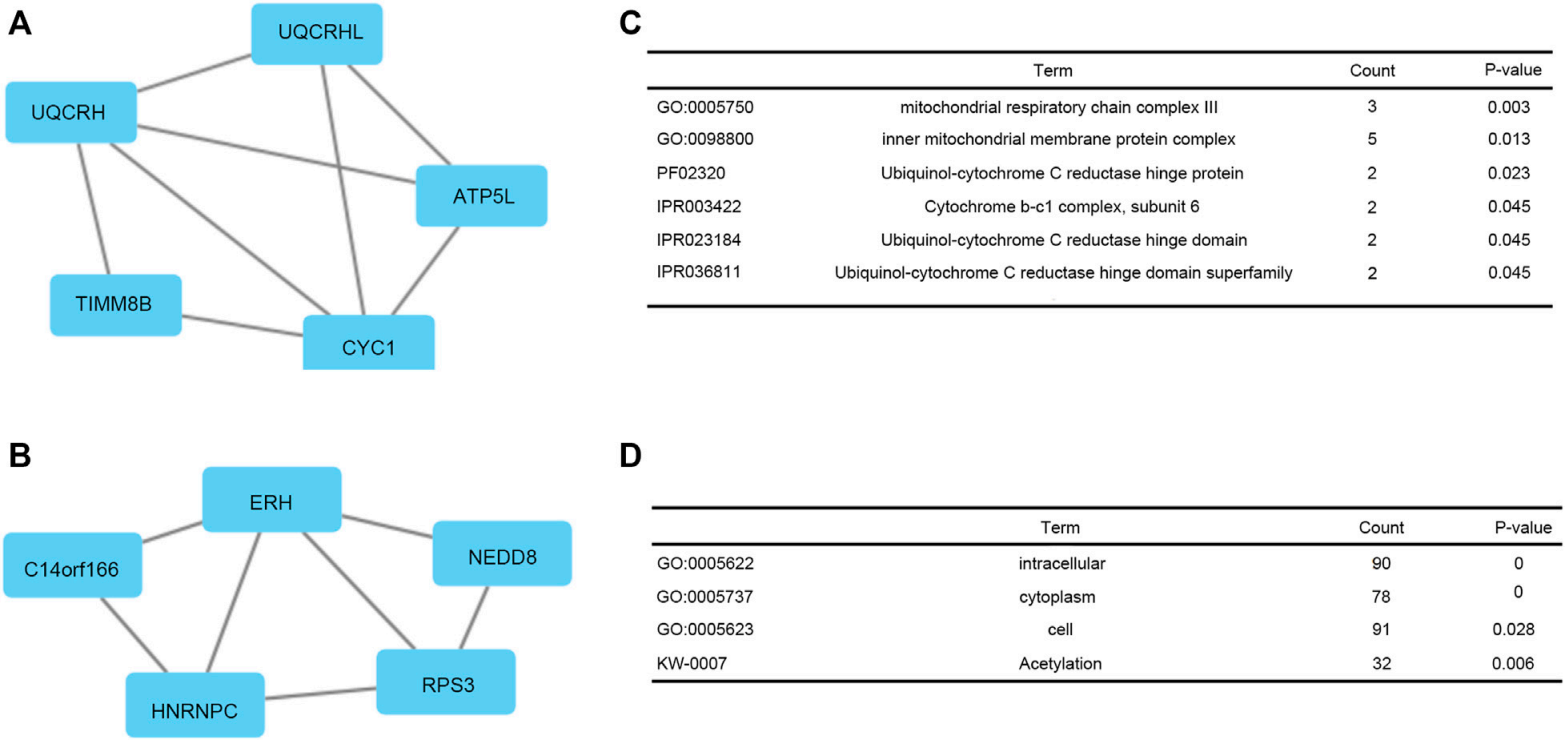
Top two modules from the PPI network. **(A, C)** PPI network and enrichment analysis of module 1. **(B, D)** PPI network and enrichment analysis of module 2.

PPI network of co-expressed genes conforming to the STRING conditions was assembled and visualized by Cytoscape (Figures 7A,B). The top two modules were screened out and conducted GSEA (Figures 7C,D). The GSEA indicated that genes were related to GO:0005750 mitochondrial respiratory chain complex III, GO:0098800 inner mitochondrial membrane protein complex, PF02320 Ubiquinol-cytochrome C reductase hinge protein, IPR003422 Cytochrome b-c1 complex, subunit 6, IPR023184 Ubiquinol-cytochrome C reductase hinge domain, IPR036811 Ubiquinol-cytochrome C reductase hinge domain superfamily, GO:0005622 intracellular, GO:0005737 cytoplasm, GO:0005623 cell, and KW-0007 acetylation.

### Relationship Between RAB11FIP1 Expression and Tumor-Infiltrating Immune Cells

**FIGURE 8:**
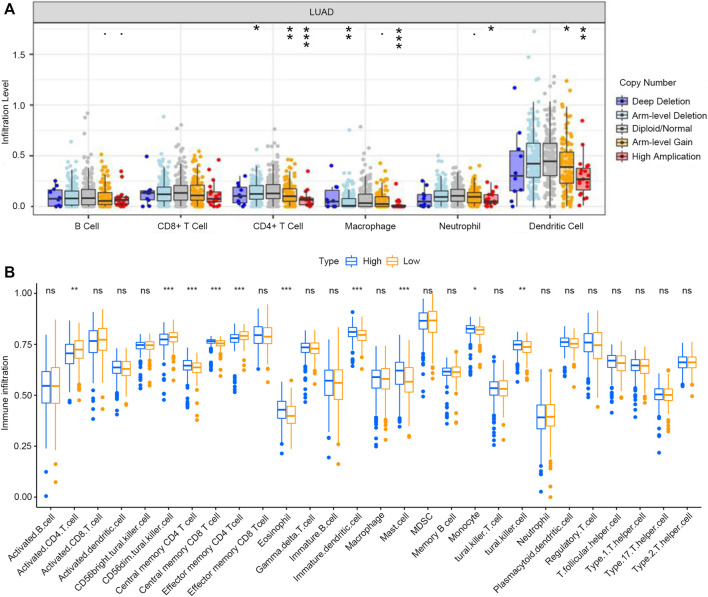
Relationship between expression of RAB11FIP1 and level of immune invasion in LUAD **(A)** RAB11FIP1 copy number variation affected the infiltration level of CD8^+^ T cells, Macrophages, Neutrophils, and Dendritic cells in lung tissues. **(B)** The distribution of 28 subtypes of immune cells in low and high RAB11FIP1 expression groups.

The TIMER database was used to investigate whether the expression of RAB11FIP1 in lung cancer was related to the level of immune invasion. The results suggested that the copy number variation (CNV) of RAB11FIP1 was significantly related to the infiltration levels of CD4^+^ T cells, macrophages, neutrophils, and dendritic cells (Figure 8A). Then, the differences of 28 types of TIICs between the RAB11FIP1 low-expression and RAB11FIP1 high-expression groups were compared. The results indicated that the low-expression group had more activated CD4 T cell (*p* < 0.01), CD56 dim natural killer cell (*p* < 0.001), effector memory CD4 T cell (*p* < 0.001) and fewer central memory CD4 T cell (*p* < 0.001), central memory CD8 T cell (*p* < 0.001), eosinophil (*p* < 0.001), immature dendritic cell (*p* < 0.001), mast cell (*p* < 0.001), natural killer cell (*p* < 0.01), and monocyte (*p* < 0.05) infiltrates. Other TIICs showed no statistically significant intergroup differences (Figure 8B). Meanwhile, the correlation between RAB11FIP1 expression and the diverse marker genes of TIICs by the TIMER database was further investigated. After adjustment of the purity (Table 3), the marker genes KIR2DL4 and KIR3DL3 of natural killer cells, IFNG of Th1, and GZMB of T cell exhaustion had a negative relation with RAB11FIP1, with a positive correlation with all TAM, M1 macrophage, M2 macrophage, neutrophil, dendritic cell, Th2, and Treg marker genes. RAB11FIP1 expression was also positively related to some marker genes of monocyte, Th1, Tfh, Th17, and T cell exhaustion. To verify the above conclusions, we explored the relation between RAB11FIP1 expression and CD8 + T cell, T cell (general), B cell, TAM, M1 macrophage, M2 macrophage, and Treg cell marker genes in GEPIA (Table 4). The results indicated that RAB11FIP1 expression had positive relevance to TAM, M1 macrophage, M2 macrophage, and Treg genes.

**TABLE 3:** Correlation analysis between RAB11FIP1 and marker genes of immune cells in TIMER.

Description	Marker genes	None Cor	P	Purity Cor	P
CD8 + T cell	CD8A	0.025	0.568	0.011	0.812
	CD8B	0.002	0.964	−0.018	0.686
T cell (general)	CD3D	−0.040	0.361	−0.072	0.111
	CD3E	0.062	0.159	0.051	0.261
	CD2	0.047	0.284	0.031	0.493
B cell	CD19	−0.010	0.829	−0.032	0.480
	CD79A	−0.021	0.639	−0.037	0.408
	CD27	−0.006	0.893	−0.023	0.607
Monocyte	CD14	0.063	0.154	0.054	0.228
	CD86	0.146	0.001	0.142	0.002
	CSF1R	0.234	7.49E–08	0.240	7.03E–08
TAM	CD68	0.178	5.03E-05	0.181	5.29E–05
M1 Macrophage	NOS2	0.144	0.001	0.135	0.003
	IRF5	0.276	1.81E-10	0.277	3.70E–10
	PTGS2	0.133	0.002	0.120	0.008
M2 Macrophage	CD163	0.197	6.35E-06	0.198	9.78E–06
Neutrophil	CEACAM8	0.260	2.07E–09	0.269	1.34E-09
	ITGAM	0.253	5.80E–09	0.268	1.56E–09
	CCR7	0.170	0	0.171	0
Natural killer cell	KIR2DL1	0.012	0.779	0.002	0.959
	KIR2DL3	-0.003	0.939	-0.003	0.950
	KIR2DL4	−0.104	0.018	−0.126	0.005
	KIR3DL1	0.054	0.223	0.036	0.425
	KIR3DL2	0.008	0.852	0	0.996
	KIR3DL3	−0.096	0.030	−0.100	0.027
	KIR2DS4	0.045	0.306	0.040	0.373
Dendritic cell	CD1C	0.227	1.81E–07	0.231	2.10E–07
	NRP1	0.215	8.24E–07	0.214	1.59E–6
	ITGAX	0.238	4.61E–08	0.253	1.16E–08
Th1	TBX21	0.093	0.035	0.089	0.049
	STAT4	0.113	0.010	0.099	0.027
	IFNG	−0.138	0.002	−0.157	0.000
	TNF	0.090	0.041	0.076	0.094
	STAT1	0.079	0.074	0.059	0.190
Th2	GATA3	0.131	0.003	0.130	0.004
	STAT5A	0.267	7.23E–10	0.275	5.35E–10
	STAT6	0.285	4.69E–11	0.295	2.54E–11
Tfh	BCL6	0.306	1.18E–12	0.299	1.24E–11
	IL21	0.012	0.789	0.002	0.968
Th17	STAT3	0.335	6.09E–15	0.332	3.95E–14
	IL17A	−0.014	0.748	−0.031	0.492
Treg	FOXP3	0.117	0.008	0.111	0.014
	CCR8	0.149	0.001	0.144	0.001
	TGFB1	0.256	3.76E-09	0.255	9.05E-09
	STAT5B	0.309	7.64E-13	0.307	3.49E-12
T cell exhaustion	PDCD1	0.016	0.714	−0.002	0.961
	CTLA4	0.061	0.168	0.048	0.292
	LAG3	0.005	0.917	−0.009	0.834
	HAVCR2	0.134	0.002	0.131	0.004
	GZMB	−0.132	0.003	−0.160	0

TAM, tumor-associated macrophage; Th, T helper cell; Tfh, Follicular helper T cell; Treg, regulatory T cell; Cor, R-value of Spearman’s correlation; None, correlation without adjustment. Purity, correlation adjusted by purity.

**TABLE 4:** Correlation analysis between RAB11FIP1 and TIICs marker genes in GEPIA.

Description	Marker genes	Tumor R	P	Normal R	P
CD8 + T cell	CD8A	0.069	0.130	−0.016	0.900
	CD8B	0.045	0.320	−0.18	0.180
	CD45	0.320	3.5e−13	0.200	0.120
T cell (general)	CD3D	−0.034	0.450	−0.34	0.008
	CD3E	0.094	0.039	−0.062	0.640
	CD2	0.086	0.058	−0.16	0.210
B cell	CD19	−0.022	0.620	−0.21	0.120
	CD79A	−0.037	0.420	−0.19	0.160
	CD27	−0.013	0.770	−0.28	0.034
TAM	CD68	0.340	6.2e−15	−0.053	0.690
	CD11B	0.370	5.5e−17	0.060	0.650
M1 Macrophage	NOS2	0.200	5.8e−06	0.450	4e−04
	IRF5	0.310	2.3e−12	−0.2	0.130
	PTGS2	0.160	0.001	0.500	6.3e−05
M2 Macrophage	CD163	0.220	1.3e−06	−0.061	0.650
	CD206	0.400	2.2e−20	0.140	0.290
Treg	FOXP3	0.160	0.000	−0.026	0.850
	CCR8	0.280	4e−10	0.020	0.880
	TGFB1	0.280	2.3e−10	−0.024	0.850
	STAT5B	0.430	7.8e−23	0.570	2.5e−06

TAM, Tumor-associated macrophages; Treg, regulatory T cell. Tumor, correlation analysis in tumor tissue of TCGA. Normal, correlation analysis in normal tissue of TCGA.

## Discussion

RAB11FIP1 (also known as Rab-coupling protein, RCP), a member of the Rab11-family interacting proteins (Rab11-FIP), has an effect on the Rab-11 mediated recycling of vesicles and is involved in endosomal trafficking and receptor sorting (Jing et al., 2010; Rainero et al., 2012; Baetz and Goldenring, 2013), including trafficking of integrin α5β1 which is a receptor for fibronectin and helpful in cancer cell invasion, metastasis, and withstanding anticancer drugs (Morello et al., 2011; Paul et al., 2015). Over the past few years, the abnormal RAB11FIP1 expression has been discussed in different strains of malignancies. Although RAB11FIP1 has been considered a crucial role in the formation and development of many cancers for some time past, its role in LUAD is still not clear.

A previous study noted that RAB11FIP1 is a multifunctional gene frequently amplified in breast cancer and low-expressed at the early stage of cervical cancer (Zhang et al., 2009; Zhang et al., 2016). Meanwhile, overexpression of miR-93 via targeting RAB11FIP1 as an early event plays an important role in the oncogenesis of cervical cancer (Zhang et al., 2016). In esophageal cancer, RAB11FIP1 regulates EMT by directly inhibiting the key transcription factor ZEB1 of EMT. Recent findings suggest that RAB11FIP1 regulates organoid formation, tumor cell invasion, and EMT (Tang et al., 2021). In the current study, we assured that RAB11FIP1 could be a prognostic biomarker. Through data mining, we investigated RAB11FIP1 expression levels in LUAD, which were identified from the Oncomine database. We found that the expression of RAB11FIP1 in tumor tissues was lower than normal adjacent tissues in patients with LUAD. We then compared RAB11FIP1 expression in LUAD tissues with that in adjacent normal tissues using the UALCAN database and IHC and the results also showed that the RAB11FIP1 expression was related to disease stage and tumor size in LUAD patients. Furthermore, the overall survival (OS) of patients with lung cancer in the low RAB11FIP1 expression group compared with those in the high RAB11FIP1 expression group was significantly shorter (*p* < 0.05). The univariate Cox analysis showed that RAB11FIP1, age, and sex were associated with OS, while the multivariate analysis showed that age and RAB11FIP1 had independent prognostic values, all suggesting that RAB11FIP1 is a prognostic biomarker of LUAD.

To figure out the control mechanism for LUAD, we explored the protein-coding genes correlated with RAB11FIP1 and its co-expression genes in LUAD tissues. The proteins related to RAB11FIP1 were involved in mitochondrial respiration and mitochondrial membrane synthesis. GO and KEGG enrichment analyses indicated that the disulfide oxidoreductase activity, electron transport chain, mitochondrial protein complex, ribosome, and oxidative phosphorylation were most negatively correlated with the expression of RAB11FIP1; Ras guanyl nucleotide exchange factor activity, non-small cell lung cancer, T cell receptor signaling pathway, TGF beta signaling pathway, and B cell receptor signaling pathway were most negatively correlated with the expression of RAB11FIP1, so we speculated that it might be a party to cell proliferation and apoptosis. Previous research has also reported that down-regulation of RAB11FIP1 can promote the proliferation of cervical cancer cells and inhibit cell apoptosis (Zhang et al., 2016). This result helps to explore the biological role played by RAB11FIP1 in LUAD.

Immune cells have essential auxiliary functions and influence clinical outcomes in cancer (Sui et al., 2020). In this study, we disclosed that different immune infiltration levels of LUAD could induce different expression levels of RAB11FIP1. CIBERSORT algorithm was utilized to conclude that a low RAB11FIP1 expression was associated with decreased infiltrates of central memory CD4 T cell, centralr memory CD8 T cell, eosinophil, immature dendritic cell, mast cell, natural killer cell, and monocyte, and increased infiltrates of activated CD4 T cell, CD56 dim natural killer cell, and effector memory CD4 T cell. In addition, TIMER and GEPIA databases indicated a correlation between gene markers of different immune cells and RAB11FIP1 expression. This might show that RAB11FIP1 regulated the tumor immune microenvironment of LUAD. Within the tissues, immature DCs act as sentinels, sounding the alarm for signs of tissue damage or infection. Importantly, DCs are essential to initiate an anti-cancer immune response as they can also detect tumor antigens produced by cancer cells, such as mutated or aberrantly expressed proteins (de Winde et al., 2020). Meanwhile, the differentiation of CD4^+^ T cells is critical for productive antitumor responses (Binnewies et al., 2019). In our study, the RAB11FIP1 low expression group showed more activated CD4 T cells, effector memory CD4 T cells, and fewer central memory CD4 T cells, and the expression of RAB11FIP1 was positively correlated with the overall level of CD4 T cells. Hence, we deduced that RAB11FIP1 expression mainly reduced central memory CD4 T cells. Meanwhile, immature dendritic cell infiltrates were low in the RAB11FIP1 low expression group. We suspected that the decrease of DC reduced the activation of initial T cells by secreting cytokines and costimulatory molecules (Binnewies et al., 2019). Through the above analysis, we found that the expression of RAB11FIP1 was positively correlated with DC and CD4 T cells, suggesting a poor prognosis for LUAD.

## Conclusion

RAB11FIP1 could be regarded as an early diagnostic and independent prognostic indicator for LUAD patients. However, this research has several limitations. The main limitation was that our data were selected from databases, so sample contents might be insufficient. In addition, the possible mechanisms of the involvement of RAB11FIP1 in the tumorigenesis and progression of LUAD remain to be further investigated.

## Data Availability

The datasets presented in this study can be found in online repositories. The names of the repository/repositories and accession number(s) can be found in the article/Supplementary Material.
