# Supplementary material for: RAB11FIP1: An Indicator for Tumor Immune Microenvironment and Prognosis of Lung Adenocarcinoma from a Comprehensive Analysis of Bioinformatics
Source: Front Genet. 2021 Oct 26;12:757169. doi: 10.3389/fgene.2021.757169 (PMC8576257; doi:10.3389/fgene.2021.757169)
Supplement: Supplementary file 1 [file DataSheet1.docx]

**Supplementary Table 1 | GO annotation for significant enrichment of RAB11FIP1 in lung cancer cells (Biological processes) (LinkedOmics)**

| **Description** | **Leading EdgeNum** | **FDR** | **Leading Edge Gene** |
| --- | --- | --- | --- |
| regulation of small GTPase mediated signal transduction | 125 | 0 | A2M;ABCA1;ABR;ADRA1A;AKAP13;AMOT;ARAP1;ARAP2;ARAP3;ARFGEF1;ARHGAP1;ARHGAP12;ARHGAP20;ARHGAP23;ARHGAP24;ARHGAP29;ARHGAP30;ARHGAP31;ARHGAP32;ARHGAP42;ARHGAP6;ARHGAP8;ARHGEF10L;ARHGEF11;ARHGEF12;ARHGEF15;ARHGEF16;ARHGEF17;ARHGEF37;ARHGEF38;ARHGEF5;ARHGEF6;ARHGEF7;ARHGEF9;ARRB1;AUTS2;BCL6;BCR;CBL;CD2AP;CGNL1;CYTH1;CYTH3;DAB2IP;DLC1;DNM2;DNMBP;ECT2L;EPS8L1;EPS8L2;F2RL1;FAM13B;FARP1;FARP2;FBP1;FGD2;FGD3;FGD4;FGD5;GARNL3;GBF1;GMIP;GPR17;GPR55;GRB2;HEG1;INPP5B;IQSEC1;IQSEC2;ITGA3;ITSN1;ITSN2;KALRN;KANK1;KANK2;LYN;MADD;MAPRE2;MCF2L;MFN2;MYO9A;MYO9B;MYOC;NET1;NF1;NOTCH1;NOTCH2;OPHN1;PLCE1;PLEKHG1;PLEKHG7;PPP2CB;PREX2;PSD3;PSD4;RALGAPA2;RALGAPB;RALGPS1;RAP1GAP;RAPGEF1;RASA2;RASGRF1;RASGRP1;RASGRP4;RHOBTB2;RTN4R;SCAI;SIPA1L2;SIPA1L3;SLIT2;SOS1;SPATA13;SPRY1;SPRY2;SRC;SRGAP2;SRGAP3;STARD13;STARD8;SYDE2;TGFB2;TIAM1;TNK1;TRIO;TSC2 |
| cell junction organization | 107 | 0 | ACE;ACE2;ACTN2;ACTN4;ACVRL1;ADD1;APC;APOD;ARHGAP6;ARHGEF7;ARVCF;BCAS3;CADM1;CADM3;CD9;CDH1;CDH19;CDH20;CDH5;CLDN1;CLDN3;CLDN9;COL17A1;CRB3;CSF1R;CTNNA1;CTNND1;CXADR;DLC1;DST;DUSP3;EPHA2;F11R;F2RL1;FAM107A;FBF1;FLCN;FLNA;FMN1;FZD5;GJB1;GRHL1;GRHL2;HEG1;HIPK1;IKBKB;IQGAP1;IQSEC1;ITGA2;ITGB4;JUP;KDR;LDB1;LIMCH1;LIMS2;LSR;MACF1;MAPRE2;MARVELD2;MARVELD3;MYADM;MYO1C;MYO9A;MYOC;NFASC;NPHP4;OCLN;OPHN1;PARD6B;PDCD6IP;PDPK1;PIP5K1C;PKP4;PLEC;PLEKHA7;PRKCA;PRKCH;PTK2B;PTPN23;PTPRJ;PTPRK;RAPGEF1;RAPGEF2;ROCK1;ROCK2;RUNX1;SDC4;SDK2;SLC9A1;SLK;SMAD3;SMAD7;SORBS1;SRC;STRN;TAOK2;TBCD;TEK;TGFB1;TGFB2;TJP1;TLN1;TNS1;TSC1;VCL;WHAMM;ZNF703 |
| cell-cell adhesion via plasma-membrane adhesion molecules | 92 | 0 | ACVR1;ALCAM;AMIGO1;ANXA3;ARVCF;CADM1;CADM3;CD84;CDH1;CDH19;CDH20;CDH23;CDH5;CDHR1;CDHR4;CEACAM1;CEACAM5;CEACAM6;CEACAM8;CELSR1;CELSR2;CLDN1;CLDN16;CLDN18;CLDN2;CLDN20;CLDN23;CLDN3;CLDN4;CLDN8;CLDN9;CLSTN1;CNTN2;CNTN4;CNTN6;CX3CL1;CXADR;DCHS2;DSCAML1;EMB;ESAM;FAT2;FAT3;FAT4;GATA5;GPC4;HMCN1;ICAM1;IGSF9B;IL1RAP;ITGAL;ITGAM;ITGB2;LRRC4;LRRC4C;MBP;MMP24;MPZL2;MYADM;PCDH1;PCDH12;PCDH15;PCDH17;PCDH19;PCDH20;PCDHA10;PCDHA3;PCDHAC2;PCDHGA10;PCDHGA12;PCDHGA5;PCDHGA6;PCDHGA9;PCDHGB6;PCDHGB7;PIK3CB;PKD1;PLXNB2;PLXNB3;PTPN23;PTPRD;PTPRF;PTPRG;PTPRT;ROBO2;ROBO4;SDK2;SELP;SPARCL1;TGFB2;TGFBR2;WNK1 |

**Abbreviations: LeadingEdgeNum, the number of leading-edge genes; FDR, false discovery rate from Benjamini and Hochberg from gene set enrichment analysis (GSEA).**

**Supplementary Table 2 | GO annotation for significant enrichment of RAB11FIP1 in lung cancer cells (Cellular components) (LinkedOmics)**

| **Description** | **Leading EdgeNum** | **FDR** | **leadingEdgeGene** |
| --- | --- | --- | --- |
| cell-cell junction | 135 | 0 | ACTN4;ADD1;AHNAK;AKAP6;AMOT;AMOTL2;ANK3;APC;ARVCF;ASH1L;ATP1A1;ATP1A2;BAIAP2;BMPR2;CADM3;CASK;CAV3;CCND1;CD2AP;CDC42BPA;CDC42EP1;CDC42EP4;CDH1;CDH19;CDH5;CDSN;CEACAM1;CGN;CGNL1;CLDN1;CLDN16;CLDN18;CLDN20;CLDN23;CLDN3;CLDN4;CLDN8;CLDN9;CNKSR1;COL13A1;COL17A1;CTNNA1;CTNND1;CXADR;CYTH1;CYTH3;DAG1;DDX6;DES;DLG3;DPP4;EPB41L4B;EPHA2;EPPK1;ESAM;EVPL;F11R;FAT2;FLNA;FRMD4B;FXYD1;FZD5;GJB1;GRHL2;HEG1;HMCN1;ILDR1;IQGAP1;JUP;KCNA5;KLHL24;LIMD1;LSR;LYN;MAGI1;MAGI2;MAGI3;MARVELD2;MARVELD3;MLC1;MYADM;MYH9;NFASC;NPHP4;OCLN;P2RX7;PACSIN2;PARD3B;PARD6B;PCDH1;PCDHGA12;PDCD6IP;PDZD2;PGM5;PIK3R1;PIKFYVE;PKD2;PKP4;PLEKHA7;PPL;PRKCD;PRKCZ;PTK7;PTPRJ;PTPRK;PTPRU;RAP2B;RAPGEF2;SCN1A;SCN4B;SGCA;SHROOM2;SHROOM3;SHROOM4;SIPA1L3;SLC9A1;SMAD7;SORBS1;STRN;STX3;SYNPO;TACSTD2;TBCD;TEK;TIAM1;TJP1;TJP2;TJP3;TNKS1BP1;TRPC6;UBN1;USP53;VCL;WASF2;WTIP |
| basolateral plasma membrane | 75 | 0 | ABCC6;ADAM9;ADRA2A;ANK3;AQP1;AQP4;AQP5;ARRB1;ATP1A1;ATP7A;BMPR2;CA4;CASK;CD300LG;CD34;CEACAM1;CEACAM5;CLDN1;CLDN4;CLDN8;CNNM4;CXADR;DAG1;DISP1;DLG3;DST;DSTYK;EPPK1;ERBB2;ERBB3;ERBB4;EZR;FOLR1;GPIHBP1;HPGD;IL6R;ITGA3;ITGA9;KCNQ1;KCNQ4;LDLR;LDLRAP1;LRP1;MAP7;MARVELD2;MEGF11;MLC1;MUC20;MYO1C;MYO1D;NOD1;P2RY1;P2RY12;PALM;PKD1;PKD2;PROM2;PTH1R;SHROOM4;SLC12A6;SLC14A1;SLC26A5;SLC29A1;SLC40A1;SLC41A1;SLC4A4;SLC7A6;SLC9A1;SLCO4C1;ST14;STK39;TACSTD2;TEK;TJP1;VSIG1 |
| lateral plasma membrane | 20 | 0 | ABCC6;ANK3;APC;CDH1;CEACAM1;CLDN1;CLDN3;CLDN4;ERBB3;GJB1;IQGAP1;JUP;MARK2;MYO1C;NUMA1;OCLN;PKD1;SLC26A5;TACSTD2;TBCD |
| apical part of cell | 99 | 0 | ABCC6;ADRB2;AGER;AHCYL1;AMOTL2;ANO1;AQP1;AQP5;ATP1A1;ATP1B2;ATP6V0D2;ATP7A;BMPR2;CA4;CACNB3;CD300LG;CD34;CD9;CEACAM1;CEACAM5;CEACAM6;CFTR;CLCN5;CLDN1;CLDN4;CNKSR3;DPP4;DSTYK;DUOX1;DUOX2;EMP2;ENPP3;EPB41L4B;ERBB2;ERBB3;EZR;FAT4;FOLR1;FXYD1;GPIHBP1;IL6R;ITGA8;ITPR3;KCNA5;KCNE1;KL;LDLR;LMO7;MAL2;MARVELD2;MGAM;MLC1;MUC1;MUC20;MYO1B;MYO5B;MYO6;NOD1;NOTCH1;OCLN;P2RX2;P2RY1;PARD3B;PARD6B;PCM1;PLAT;PRKCZ;PROM1;PROM2;PTH1R;RAB27A;RAB27B;RAPGEF2;SCNN1A;SCNN1B;SHANK2;SHROOM2;SHROOM3;SHROOM4;SIPA1L3;SLC12A2;SLC1A1;SLC26A9;SLC29A1;SLC34A2;SLC44A4;SLC46A1;SLC5A8;SLC6A20;SLC9A1;SLC9A3R2;SPTBN2;STK39;STX3;TEK;TJP1;TNIK;VASH1;ZMYND10 |
| basal part of cell | 21 | 0 | ANK3;AQP1;AQP5;BMPR2;CD34;CEACAM1;CLDN4;DST;EDN1;ERBB2;ERBB3;ERBB4;ITGA9;LDLRAP1;MUC20;MYO1C;PHLDB1;PKD2;SHROOM4;TACSTD2;TEK |

**Abbreviations: LeadingEdgeNum, the number of leading-edge genes; FDR, false discovery rate from Benjamini and Hochberg from gene set enrichment analysis (GSEA).**

**Supplementary Table 3 | GO annotation for significant enrichment of RAB11FIP1 in lung cancer cells (Molecular functions) (LinkedOmics)**

| **Description** | **Leading EdgeNum** | **FDR** | **leadingEdgeGene** |
| --- | --- | --- | --- |
| guanyl-nucleotide exchange factor activity | 140 | 0 | ABR;ACTN2;ADRB1;AKAP13;ALS2CL;ANGPT1;ARFGEF1;ARHGEF10L;ARHGEF11;ARHGEF12;ARHGEF15;ARHGEF16;ARHGEF17;ARHGEF18;ARHGEF37;ARHGEF38;ARHGEF5;ARHGEF6;ARHGEF7;ARHGEF9;BCR;BTC;CAMK2D;CAMK2G;CSF2RA;CYTH1;CYTH3;DENND1C;DENND2C;DENND3;DENND4A;DENND4C;DLG2;DLG3;DNMBP;DOCK10;DOCK11;DOCK2;DOCK3;DOCK6;DOCK8;DOCK9;ECT2L;EPS8;EPS8L1;EPS8L2;ERBB2;ERBB3;ERBB4;FARP1;FARP2;FGD2;FGD3;FGD4;FGD5;FGD6;FGF10;FGF20;FGFR2;FGFR3;FLCN;FLT3;FNIP1;FYN;GAPVD1;GBF1;GFRA1;GFRA2;GRB2;GRIN2A;HBEGF;HERC1;HERC2;HGF;HPS1;HPS4;IL3RA;IL5RA;IQSEC1;IQSEC2;IQSEC3;IRS1;ITSN1;ITSN2;JAK1;KALRN;KL;KNDC1;MADD;MCF2L;MYCBP2;NET1;P2RY12;PDGFA;PLCE1;PLEKHG1;PLEKHG6;PLEKHG7;PREX2;PSD3;PSD4;PTGIR;PTPRA;RAB3GAP1;RALGDS;RALGPS1;RAPGEF1;RAPGEF2;RAPGEF4;RAPGEF5;RAPGEF6;RAPGEFL1;RASGEF1B;RASGRF1;RASGRP1;RASGRP2;RASGRP4;RGL1;RGL3;RIN2;RIN3;RPGR;SBF1;SH2D3C;SHC2;SMCR8;SOS1;SPATA13;SPTAN1;SPTB;SPTBN1;SPTBN2;SPTBN5;ST5;TBC1D10A;TEK;TIAM1;TRAPPC10;TRIO;VAV1 |
| Rho GTPase binding | 70 | 0 | ABI2;ABR;AKAP13;ARHGDIB;ARHGEF10L;ARHGEF11;ARHGEF12;ARHGEF15;ARHGEF16;ARHGEF17;ARHGEF18;ARHGEF37;ARHGEF38;ARHGEF5;ARHGEF6;ARHGEF7;ARHGEF9;ATP7A;BCR;C15orf62;CDC42EP1;CDC42EP3;CDC42EP4;CDKL5;DAAM1;DAAM2;DIAPH1;DNMBP;DOCK11;DOCK2;DOCK3;ECT2L;EPS8L1;EPS8L2;FARP1;FARP2;FGD2;FGD3;FGD4;FGD5;FLNA;IQGAP1;IQGAP2;ITSN1;ITSN2;KALRN;KIF3B;LRRK2;MAP3K11;MCF2L;MTSS1L;MYO9B;NET1;PARD6B;PLEKHG1;PLEKHG6;PLEKHG7;PREX2;RASGRF1;ROCK1;ROCK2;SOS1;SPATA13;SRGAP2;SRGAP3;TIAM1;TRIO;TRIOBP;VAV1;WHAMM |

**Abbreviations: LeadingEdgeNum, the number of leading-edge genes; FDR, false discovery rate from Benjamini and Hochberg from gene set enrichment analysis (GSEA).**

**Supplementary Table 4 | KEGG annotation for significant enrichment of RAB11FIP1 in lung cancer cells (LinkedOmics)**

| **Description** | **Leading EdgeNum** | **FDR** | **leadingEdgeGene** |
| --- | --- | --- | --- |
| Cell adhesion molecules (CAMs) | 63 | 0 | ALCAM;CADM1;CADM3;CD22;CD28;CD34;CD4;CD40LG;CDH1;CDH5;CLDN1;CLDN16;CLDN18;CLDN2;CLDN20;CLDN23;CLDN3;CLDN4;CLDN8;CLDN9;CNTN1;CNTN2;CNTNAP1;ESAM;F11R;HLA-DMA;HLA-DOA;HLA-DPA1;HLA-DPB1;HLA-DQA1;HLA-DQB1;HLA-DRA;HLA-DRB1;HLA-DRB5;HLA-E;ICAM1;ICOSLG;ITGA8;ITGA9;ITGAL;ITGAM;ITGB2;ITGB8;JAM2;LRRC4;LRRC4B;LRRC4C;NCAM2;NFASC;NLGN3;NRXN2;NRXN3;NTNG1;OCLN;PTPRF;SDC1;SDC3;SDC4;SELP;SELPLG;SIGLEC1;SPN;VTCN1 |

**Abbreviations: LeadingEdgeNum, the number of leading-edge genes; FDR, false discovery rate from Benjamini and Hochberg from gene set enrichment analysis (GSEA).**
